# Learning the dynamics of realistic models of *C. elegans* nervous system with recurrent neural networks

**DOI:** 10.1038/s41598-022-25421-w

**Published:** 2023-01-10

**Authors:** Ruxandra Barbulescu, Gonçalo Mestre, Arlindo L. Oliveira, Luís Miguel Silveira

**Affiliations:** 1 INESC-ID Lisboa, Rua Alves Redol 9, Lisbon, 1000-029 Portugal; 2 IST Técnico Lisboa, Universidade de Lisboa, Av. Rovisco Pais 1, Lisbon, 1049-001 Portugal

**Keywords:** Computational neuroscience, Computational science, Computer science

## Abstract

Given the inherent complexity of the human nervous system, insight into the dynamics of brain activity can be gained from studying smaller and simpler organisms. While some of the potential target organisms are simple enough that their behavioural and structural biology might be well-known and understood, others might still lead to computationally intractable models that require extensive resources to simulate. Since such organisms are frequently only acting as proxies to further our understanding of underlying phenomena or functionality, often one is not interested in the detailed evolution of every single neuron in the system. Instead, it is sufficient to observe the subset of neurons that capture the effect that the profound nonlinearities of the neuronal system have in response to different stimuli. In this paper, we consider the well-known nematode *Caenorhabditis elegans* and seek to investigate the possibility of generating lower complexity models that capture the system’s dynamics with low error using only measured or simulated input-output information. Such models are often termed black-box models. We show how the nervous system of *C. elegans* can be modelled and simulated with data-driven models using different neural network architectures. Specifically, we target the use of state-of-the-art recurrent neural network architectures such as Long Short-Term Memory and Gated Recurrent Units and compare these architectures in terms of their properties and their accuracy (Root Mean Square Error), as well as the complexity of the resulting models. We show that Gated Recurrent Unit models with a hidden layer size of 4 are able to accurately reproduce the system response to very different stimuli. We furthermore explore the relative importance of their inputs as well as scalability to more scenarios.

## Introduction

Recent developments in experimental neuroscience have considerably increased the availability of novel recordings and reconstructions shedding further light into the structure and function of the brain as well as many other systems. But understanding the complexities behind the relations between structure and function as well as the behaviour of such systems across multiple scales in these neuronal collections is constrained by the methods available to study them. This challenge has raised interest in many related fields, such as electrophysiological analysis, imaging techniques, brain-related medicine, computational modelling, simulation, and model reduction. Many of these efforts, while not directly providing specific information regarding structural or functional dynamics, do supply large volumes of recordings, measurements or simulations of observable input-output behaviour. The availability of these large datasets raises the question of whether low complexity, data-driven, black-box models can be used to model such input-output relations with low error, avoiding the reliance on detailed inner structures that may not be known or available.

To determine whether such an approach can be used for large, complex systems, one research direction is the study of smaller and simpler nervous systems, for which the underlying principles of network organization and information processing are easier to postulate. These organisms can become useful models to gain insight into the fundaments of neuronal dynamics and whole brain organization, validate hypotheses and develop and test modelling methods, simulation instruments and model reduction techniques. The hope is that the knowledge gained from these analyses and the techniques developed for these simpler organisms can later be used to model more complex systems.

*Caenorhabditis elegans* (*C. elegans*) belongs to this category of organisms and is quickly becoming one of the benchmarks in whole brain organization studies. *C. elegans* is a nematode (roundworm) of about 1 mm in length with a compact nervous system consisting of less than 1000 cells across all sexes and around 15,000 connections^1^. This rather small nervous system allows the worm to solve basic problems such as feeding, predator avoidance and mate-finding. Moreover, at least the cell-lineage and the anatomy of *C. elegans* are invariant, in the sense that every individual possesses the same number of neurons and they occupy fixed positions in the organism; the invariance of the synaptic connections is still under debate^2^.

The relative simplicity of *C. elegans* allowed for its almost complete description from different perspectives and scales, from its genetics and genomics to the molecular biology, structural anatomy, neuronal function, circuits and behaviour. This information is available in comprehensive databases of genetics and genomics^3^, electron micrographs and associated data, online books and atlases of the neurobiology, structural and behavioural anatomy^4^. Creating a realistic model that encapsulates all this information is not a trivial task. Open-source databases of digitally reconstructed neurons^5^, computational models^6^ and collaborative solutions^7^ are opening the door for more flexible, multi-scale and multi-algorithm simulation environments for *C. elegans* and other complex biological systems.

The underlying models are based on the connectome, the map of the neuronal connections in the brain. Usually described as a neuronal network, the connectome is a graph where the nodes are the neurons and the edges represent the synapses. The complete connectome of *C. elegans* contains 302 neurons for the adult hermaphrodite^8^ and 385 neurons for the male^1^, but for the latter, the respective 3D reconstructions are not yet published. Digital reconstructions for the male are only available for the posterior nervous system of 144 neurons^9^.

The more complex the organism, the more complicated the resulting model, needing more computationally demanding and potentially intractable simulations of its dynamic behaviour. This increased complexity stems from the detailed modelling of the internal structure. However, in many cases, especially of highly complex systems, this detail is not available since the internal mechanisms may not be well known or mapped or it may be simply impossible to examine and record. Notwithstanding, frequently one is really only interested in the peripheral, or input-output behaviour, which can be checked against recorded or measured data. This motivates our efforts not only to place the focus more on observable input-output data, as well as to try and generate reduced models that avoid extraneous detail not necessary to explain these peripheral relations.

In this work we propose a methodology for generating a reduced order model of the neuronal behaviour of organisms using only peripheral information. We use *C. elegans* as a proxy for our study.

**Figure 1:**
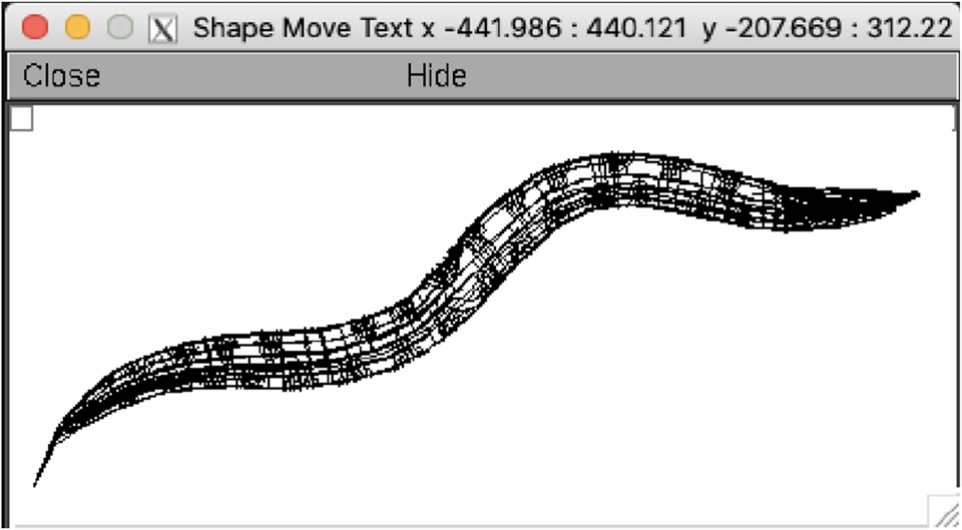
The *C. elegans* connectome described in NEURON.

Realistic models of *C. elegans*, which take into account spatial distribution and biophysical properties of neuronal compartments have been reported in the literature^10^. We start with a similar model created in-house^11^. Our model comprises the complete connectome of the adult hermaphrodite of *C. elegans*, with 302 multi-compartmental neurons and 6702 synapses^8^. The model is described in Python and implemented in NEURON^12^, one of the traditional neural simulators that has support for biologically realistic multicompartmental models of neurons. A 3D reproduction is extracted from NEURON in Fig. 1. To reproduce a certain behaviour of the worm model, stimulus is applied to the touch sensitive sensory neurons and interneurons known to be part of its corresponding circuit and we check the activity of the motor neurons and interneurons associated with that scenario. Finding strong activity in most of these neurons means that the worm performs the associated behaviour. The model was validated^11^ against four behavioural scenarios described in related literature^13^: Forward Crawling Motion (FCM) for the full network, Ablation of AVB interneurons + FCM, Ablation of AVA interneurons + FCM and the Nictation behaviour. We first reproduce here the FCM scenario, and for the purposes of this study we identified four input neurons (two sensory, two interneurons) and four output neurons (two motor, two interneurons) known to be strongly associated with forward movement^11,13^. Other dynamics can be validated similarly, and we can obtain single models that allow reproduction of multiple scenarios simultaneously^14^—see Experiment 5.

**Figure 2:**
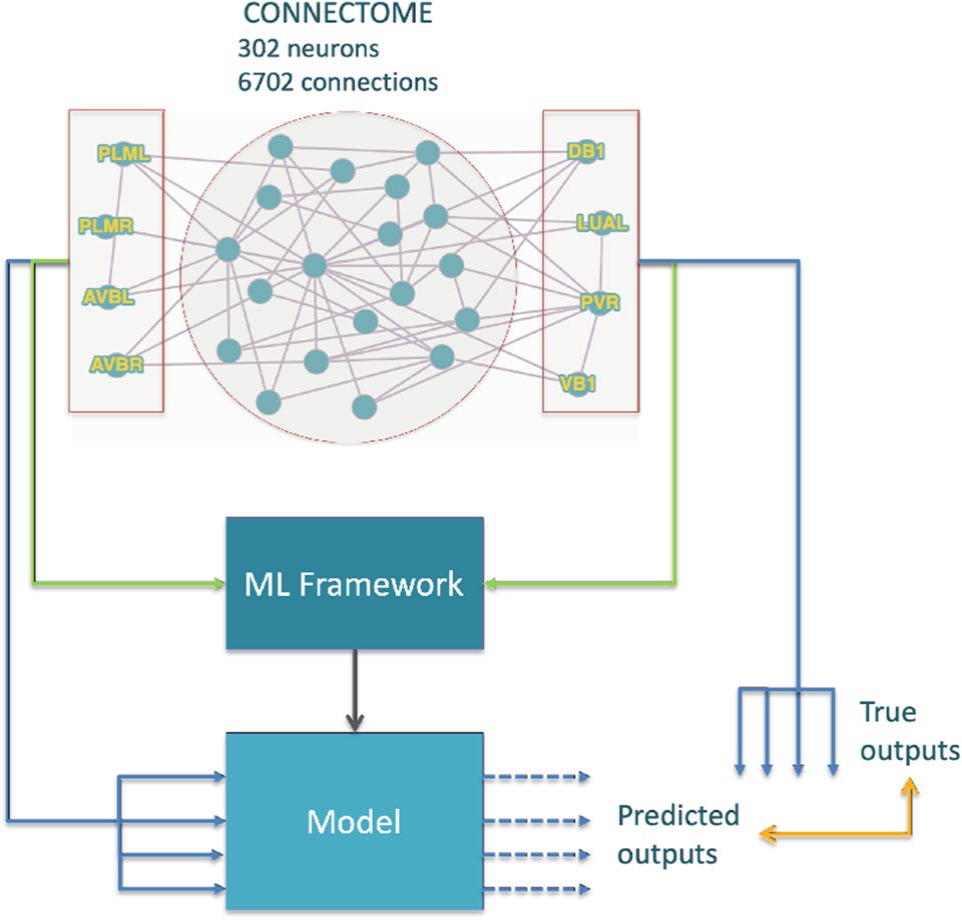
Modelling methodology. The learning machine is trained with input–output data reproducing selected behaviours and then tested against inputs it has not seen before.

Using this full 302-neuron connectome model with all its synapses, which we designate as the high-fidelity model, and the NEURON simulator, we obtain a collection of synthetic datasets representing the system’s response to different input signals. Next, assuming no prior knowledge of the original system’s structure and equations, we create a completely equation-free data-driven model using neural networks trained on these datasets. The immediate goal is to generate a reduced model to replace the original, detailed one. This reduced model should be able to reproduce with reasonably low error (the error metric used here being RMSE - Root Mean Squared Error) the behaviour of the realistic model while having fewer degrees of freedom. In this work we focus on the issue of reduced RMSE, which we equate to fidelity in reproducing the system dynamics, showing that we can produce sufficiently accurate models for analyzing the behavioural response of the *C. elegans* connectome under the described scenarios, using neural networks. The ultimate goal, however, is to show that our methodology is able to produce reduced-order, compressed models, that can be efficiently used in simulation to test and validate hypotheses regarding the behaviour and functionality of the neuronal systems of complex organisms. An illustration of our methodology is presented in Fig. 2.

## Related work and context

The connectome-based models mentioned above are often termed white-box models, as they are based on direct knowledge and access to the internal structure and parameters’ values of the modelled system. These are distributed models, where each neuron has a 3D description and position in space and the synapses are associated with neuronal sections. Such models enable highly accurate simulation of the dynamics of the systems but easily become extremely complex as they incorporate detailed structural and functional information of the system.

While the white-box approach ensures access to and evaluation of inner parameters during simulation, it has been shown that the activity of complex networks of neurons can often be described by relatively few distinct patterns, which evolve on low-dimensional manifolds^15^. This knowledge, together with the ever-present need to avoid potential numerical intractability in large-scale networks with many degrees of freedom, has generated renewed interest in applying model reduction, often also referred to as model compression, to these neuronal networks, including techniques such as Dynamic Mode Decomposition (DMD)^16^, Proper Orthogonal Decomposition (POD)^17^ and Discrete Empirical Interpolation (DEIM)^18^. Depending on the level of morphological accuracy of the underlying models, reduction techniques can have any shade of grey from white-box to black-box, the latter assuming no preliminary knowledge of the system structure and building the model solely out of knowledge of its input-output behaviour.

Black-box approaches are often built upon data-driven models, sometimes learning-based, which have the ability to grasp more naturally and more efficiently the complexity induced by the profound nonlinearities in the neuronal transmission of information. Machine-learning techniques are used to extract data-driven reduced order models for systems arising from differential equations describing the intrinsic dynamics^19^ and even to extract the governing equations of the estimated model^20^. It is therefore quite natural to consider using state of the art learning methods for developing reduced models of neuronal behaviour using data obtained from available recordings or even simulations obtained with more complex models.

Especially designed to capture temporal dynamic behaviour, Recurrent Neural Networks (RNNs), in their various architectures such as Long Short-Term Memory (LSTMs) and Gated Recurrent Units (GRUs), have been extensively and successfully used for forecasting or detecting anomalies in multivariate time series data^21–24^. Bidirectional LSTMs were used to model genome data by^25^, whereas a combination of CNNs and LSTMs generates a model for epileptic seizure recognition using EEG signal analysis in^26^. An attempt to model the human brain activity based on fMRI using RNNs (LSTMs and GRUs) is reported in^27^. In recent years, deep network approaches were used to model realistic neural activity data^28–30^. Few studies examined the behavioural output of network models of *C. elegans* using machine-learning techniques. RNNs are generated in a grey-box manner to study the chemotaxis behaviour^31^ or to predict the synaptic polarities^32^ of *C. elegans*, yet these models only include a subset of the connectome.

## Methods

Given that the starting point is in fact represented by time series data obtained from simulations of the realistic connectome-based model, the modelling task is akin to a sequence to sequence conversion for which the most suitable neural network models are the recurrent ones.

In this work we analyze the suitability of three recurrent neural networks architectures. We start with the least complex unit, the simple RNN, originally proposed in the 1980’s to model sequence data^33–35^. The second model used for the recurrent layer is the LSTM unit^36,37^, and finally we analyze its sibling, the GRU^38^.

### Recurrent neural networks

RNNs^33–35^ are a family of neural networks used for processing sequential data, particularly adept to processing a sequence of values ${\textbf {x}}^{(1)},...,{\textbf {x}}^{(t)}$, and in most cases capable to process sequences of variable length. RNNs appear from the relaxation of the condition on Feedforward Neural Networks (FFNNs) that neurons in a given layer do not form connections among themselves.

**Figure 3:**
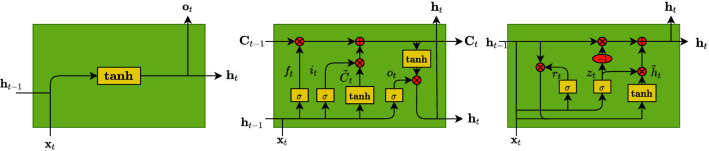
Comparison between the three different units: RNN, LSTM and GRU.

Although simple RNNs (Fig. 3-left), which are trained using Backpropagation Through Time (BPTT)^39^, seem to be a good model for sequential tasks, they are known to suffer from various issues, mainly vanishing and exploding gradients^40^. Exploding gradients refer to a large increase in the norm of the gradient during training, which appears due to the explosion of long term components that can grow exponentially faster than short term ones. This is the less common of the two problems and there are known solutions to handle it, such as the clipping gradient technique^41^. A harder to solve issue is the vanishing gradient^40^, which refers to when long term components go exponentially fast to zero, making it impossible for the model to learn the correlation between temporally distant events.

In our case, for a faithful reproduction of the dynamics, the simulations require the use of fine time steps, leading to long sequences in the datasets. This in turn implies that the response at a given time will depend on values which are far back in the sequence. This situation, however unavoidable, may lead the RNN to experience difficulties in learning our data resulting in a model with unacceptable RMSE.

### Long short-term memory

The Long Short-Term Memory unit^36^ appeared as a solution to the vanishing gradient problem, later improved with the inclusion of the forget gate to adaptively release internal resources when necessary^37^.

A LSTM unit consists of three main gates, the input gate $\textbf{i}_t$ that controls whether the cell state is updated or not, the forget gate $\textbf{f}_t$ defining how the previous memory cell affects the current one and the output gate $\textbf{o}_t$, which controls how the hidden state is updated. Note that LSTM units exhibit a major difference from RNN simple units, since besides the hidden state they also output a cell state to the next LSTM unit, as is apparent in Fig. 3-center. The LSTM mechanism is described by the following equations:

$$\begin{aligned} \textbf{i}_t&= \sigma (\mathbf {W_ix}_t + \mathbf {U_ih}_{t-1} + \mathbf {b_i}),\quad \quad \quad \tilde{\textbf{c}}_t = \phi (\mathbf {W_cx}_t + \mathbf {U_ch}_{t-1} + \mathbf {b_c}), \\ \textbf{f}_t&= \sigma (\mathbf {W_fx}_t + \mathbf {U_fh}_{t-1} + \mathbf {b_f}),\quad \quad \quad \textbf{c}_t = \textbf{f}_t \circ \textbf{c}_{t-1} + \textbf{i}_t \circ \tilde{\textbf{c}}_t, \\ \textbf{o}_t&= \sigma (\mathbf {W_ox}_t + \mathbf {U_oh}_{t-1} + \mathbf {b_o}),\quad \quad \quad \textbf{h}_t = \textbf{o}_t \circ \phi (\textbf{c}_t), \end{aligned}$$

where $\mathbf {W_i}$, $\mathbf {U_i}$, $\mathbf {W_f}$, $\mathbf {U_f}$, $\mathbf {W_o}$, $\mathbf {U_o}$, $\mathbf {W_c}$, $\mathbf {U_c}$ are weights and $\mathbf {b_i}$, $\mathbf {b_f}$, $\mathbf {b_o}$ and $\mathbf {b_c}$ are biases. All these 12 parameters are learned, while $\sigma (\cdot )$ and $\phi (\cdot )$ are the logistic sigmoid and the hyperbolic tangent activation functions, respectively. The outputs of the LSTM unit are the hidden state $\textbf{h}_t$ and the cell state $\textbf{c}_t$. The computation of the cell state requires the candidate cell state $\tilde{\textbf{c}}_t$.

### Gated recurrent units

The use of LSTM units in recurrent neural networks already produced models able to learn very distant dependencies^37^, but these units are complex structures composed of three gates. For that reason, in 2014 a new type of unit, the GRU^38^, was suggested, described by:

$$\begin{aligned} \textbf{z}_t&= \sigma (\mathbf {W_zx}_t + \textbf{U}_z\textbf{h}_{t-1} + \mathbf {b_z}), \quad \quad \quad \hat{\textbf{h}}_t = \phi (\mathbf {W_hx}_t + \mathbf {U_h}(\mathbf {r_t} \circ \textbf{h}_{t-1} + \mathbf {b_h}),\\ \textbf{r}_t&= \sigma (\mathbf {W_rx}_t + \mathbf {U_rh}_{t-1} + \mathbf {b_r}),\quad \quad \quad \textbf{h}_t = (\textbf{1} - \textbf{z}_t) \circ \textbf{h}_t + \textbf{z}_t \circ \hat{\textbf{h}}_t, \end{aligned}$$

where the weights $\mathbf {W_z}$, $\mathbf {U_z}$, $\mathbf {W_r}$, $\mathbf {U_r}$, $\mathbf {W_h}$, $\mathbf {U_h}$ and the biases $\mathbf {b_z}$, $\mathbf {b_r}$, $\mathbf {b_h}$ are the learned parameters.

The GRU (Fig. 3-right) is only composed of two gates, the update gate $\textbf{z}_t$ and the reset gate $\textbf{r}_t$. The GRU only outputs the hidden state $\textbf{h}_t$ computed based on the candidate hidden state $\hat{\textbf{h}}_t$. The update gate controls how much of the past information needs to be passed along to the future, while the reset gate is used to decide how much information the model should forget.

## Experimental setting

### Data

The starting model is based on the complete connectome of the adult hermaphrodite of *C. elegans*, with 302 multi-compartmental neurons and 6702 synapses. The neurons are described by 3D geometrical information extracted from NeuroML and LEMS files^5^ for *C. elegans*. We added membrane biophysical properties and connectivity data (chemical synapses and gap junctions) from^10^ for the complete connectome. We term this as a high-fidelity model, since due to the level of detail taken into account we assume it reproduces with fidelity the output of physical models of *C. elegans* neurons. The simulations reproduce the Forward Crawling Motion scenario, by applying varying input currents to two sensory neurons (“PLML”, “PLMR”) and two interneurons (“AVBL”, “AVBR”) and record the responses of four neurons known to have strong activity during forward locomotion (“DB1”, “LUAL”, “PVR” and “VB1”; in reality we record the responses of the entire set of neurons, but analyse only these four). The resulting system is described in Python and simulated in NEURON^12^. The Python code invokes NEURON to generate the neuronal network, simulate its behaviour with respect to certain input signals (currents) and save the responses in time of the four output neurons (voltages).

We simulate the full high-fidelity model for 500 ms with two time steps—0.5 ms and 0.1 ms—and 40 different shapes for the input currents. The input-output waveforms are extracted into two datasets of 40 snapshot files each, which are further fed to the learning framework. These datasets are available in the online repository.

**Figure 4:**
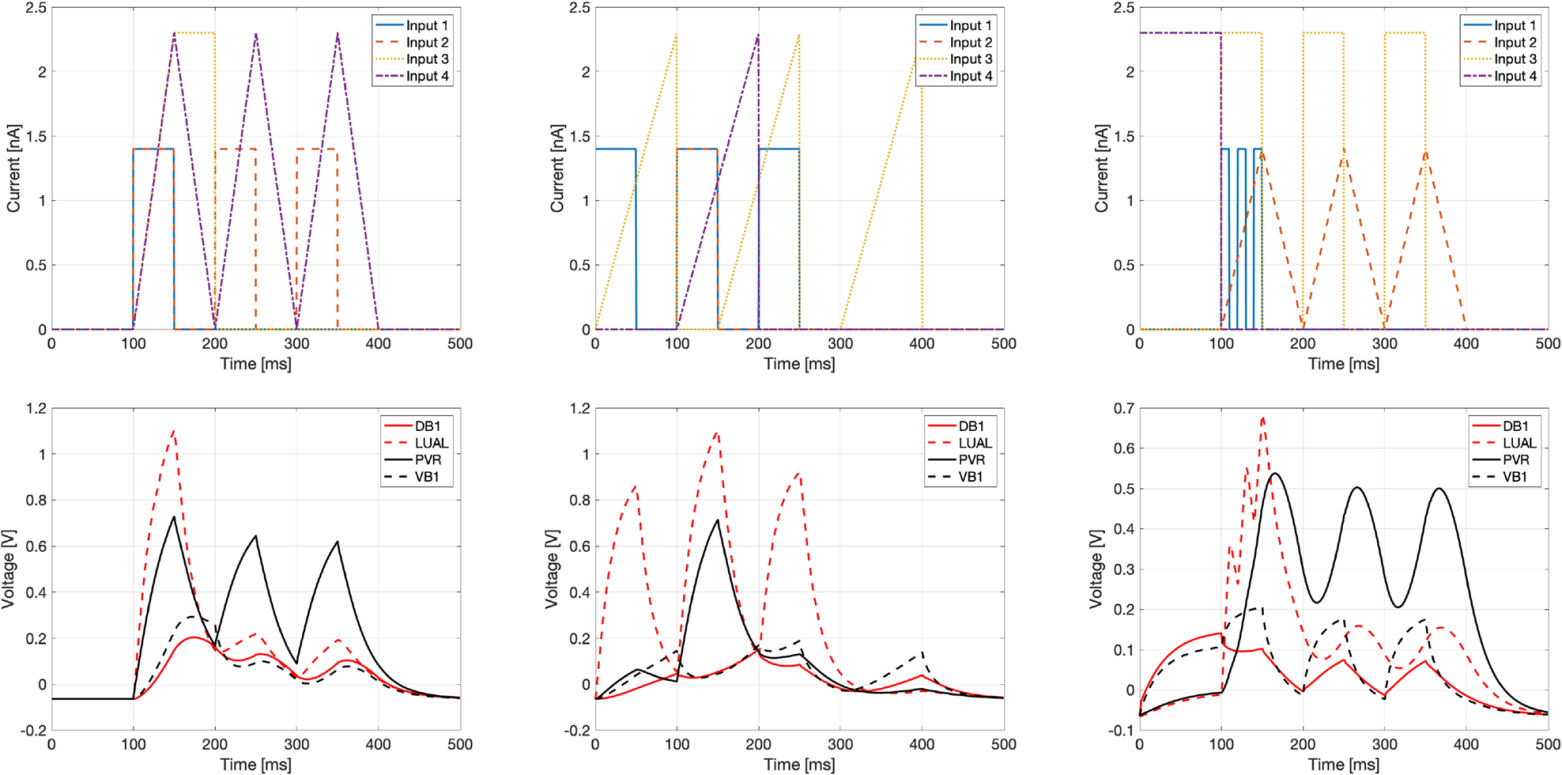
Example input (top row) and output (bottom row) time sequences.

To train and tune the hyperparameters, learning rate and batch size, the data was divided into three sets: training, validation and test. The separation of data is done as follows: the training set uses $50\%$ of the data, the validation set $25\%$ and the test set the remaining $25\%$. The separation is partially done by hand, so that validation and test sets are as diverse and demanding as possible. Alternatively, one can use an automatic separation procedure, but given the small number of sample files, visualizing the shapes was sufficient for a reliable decision for this case. Three examples from the diverse set of inputs and outputs are shown in Fig. 4.

### Modelling

The machine learning models are developed in Python^42^, using the libraries Keras^43^ and TensorFlow^44^. Details on the code and dependencies to run the experiments are listed in a Readme file available together with the code in the online repository.

All architectures consist of one recurrent layer described in “Methods” followed by a dense layer. The dense layer performs a simple linear transformation for each sequence point to convert the output of the recurrent layer, of size “hidden size”, into the four outputs.

For a consistent comparison, we fixed the optimizer to Adam^45^ and the loss function to the root mean squared error. The other two hyperparameters, the learning rate and the batch size, as well as the activation function were tuned separately for the three architectures, by keeping one fixed and varying the others, for a fixed hidden size of 16 units. Each model was trained for 1000 epochs, with the final model chosen as the best iteration on the validation set. We then fixed the hyperparameters at the optimal values yielded from this search: the batch size at 32 for all three, the activation functions kept to the default and the learning rate at 0.001 for the RNN and 0.05 for the LSTM and the GRU^14^. Note that these represent the upper limits of the learning rates, since the Adam optimizer computes individual adaptive learning rates for each parameter.

## Experiments and results

In Experiment 1 (“Experiment 1: RNN vs. LSTM vs. GRU (performance)”) we compare the performance of the three types of layers, RNN, LSTM and GRU. Experiment 2 (“Experiment 2: LSTM vs. GRU (reduction)”) carries a comparison between different sizes of the GRU recurrent layer to determine the optimal size under some RMSE constraints. Experiment 3 (“Experiment 3: GRU (long sequences)”) is an investigation upon the models’ ability to reproduce data resulting from simulations with a finer time step, therefore involving longer sequences with more data points. In Experiment 4 we investigate the relative importance of certain inputs with respect to the outputs and finally, in Experiment 5, we examine both the scalability and generalization potential of the resulting models, by significantly increasing the number of inputs and outputs, as well as mixing data from multiple scenarios. For all the experiments, the loss is computed as the average RMSE of ten runs.

**Figure 5:**
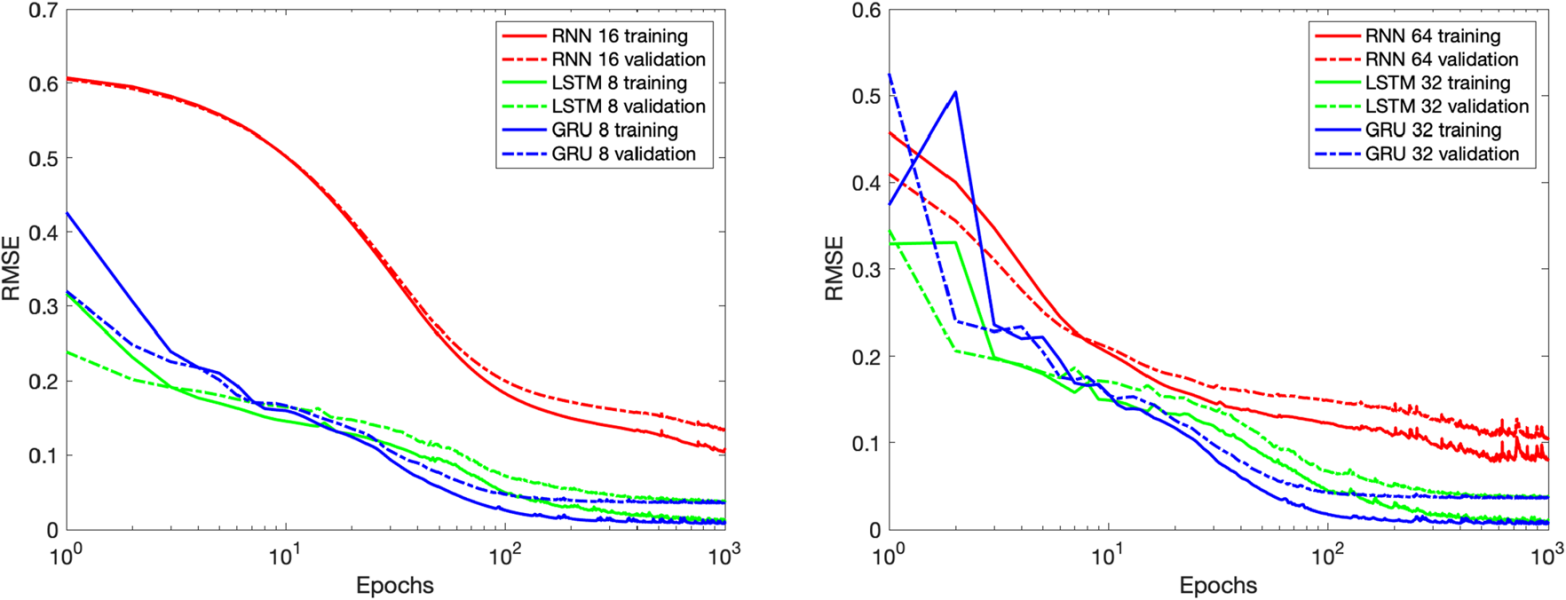
Average training and validation RMSE out of ten simulations, with recurrent layers of size 16 and 8 (left) and 64 and 32 (right).

**Figure 6:**
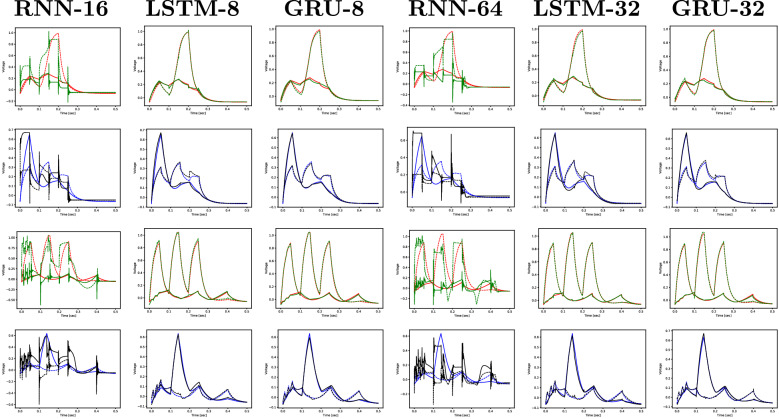
Experiment 1: realistic (red and blue) and predicted (green and black) sequences for DB1 and LUAL (1st and 3rd rows) and PVR and VB1 (2nd and 4th rows) for two sequences of the test set (one selected simulation out of ten).

### Experiment 1: RNN vs. LSTM vs. GRU (performance)

In this experiment we compare the performances of the three types of units on the dataset corresponding to the coarser time step (0.5 ms).

In the interest of fairness we use layers with comparable number of parameters, e.g. a RNN with $16$ hidden units (404 total parameters) against a LSTM and a GRU, both with $8$ units (452 and 348 parameters, respectively); and a RNN with $64$ units (4676 parameters) against a LSTM and a GRU with $32$ units (4868 and 3684 parameters, respectively).

Figure 5 shows the evolution of the training and validation losses during the training process. The simple RNN unit tends to take more time to learn, being also slightly less stable towards the end of the training process. Although this is not a good indicator, it is not as alarming as the behaviour shown in Fig. 6, where it is clear that the simple RNN unit is not able to reproduce the outputs with the desired reduced RMSE, while the LSTM and the GRU perform well. A summary of this experiment’s results is shown in Table 1. Since the simple RNN unit did not perform sufficiently well by not being able to reproduce the output with minimal RMSE, we are left with the LSTMs and GRUs units. Given that the GRU is the less complex unit of the two, we consider it the main option and keep the LSTM as an alternative architecture.

**Table 1:** The average RMSE out of ten simulations, for RNN with 16 and 64 hidden units and for LSTM and GRU with 8 and 32 hidden units, for the iteration with the smallest validation loss.

	RNN-16	LSTM-8	GRU-8	RNN-64	LSTM-32	GRU-32
Training	1.04e−01	9.93e−03	7.79e−03	6.28e−02	7.60e−03	**6.71e**−**03**
Validation	1.29e−01	3.61e−02	**3.47e**−**02**	8.87e−02	3.64e−02	3.50e−02
Test	1.34e−01	1.49e−02	1.00e−02	9.54e−02	1.52e−02	**1.23e**−**02**

Significant values are given in bold.

**Table 2:** The average RMSE of ten simulations obtained with the GRU model, for different sizes of the recurrent layer.

	2 Units	4 Units	8 Units	16 Units	32 Units	64 Units
Training	4.29e−02	1.05e−02	7.79e−03	6.78e−03	6.71e−03	**5.80e**−**03**
Validation	5.37e−02	3.48e−02	**3.47e**−**02**	3.49e−02	3.50e−02	3.62e−02
Test	6.00e−02	1.17e−02	1.00e−02	**9.36e**−**03**	1.23e−02	1.68e−02

Significant values are given in bold.

### Experiment 2: LSTM vs. GRU (reduction)

The GRU, due to its low RMSE and relative simplicity, therefore emerged as the prime candidate unit for our modelling purposes. However, we now want to determine how small the models can be without compromising the overall error. The focus of this second experiment is therefore to test different sizes of the recurrent layer and determine the smallest size that is still able to generate a model with sufficiently low RMSE.

**Figure 7:**
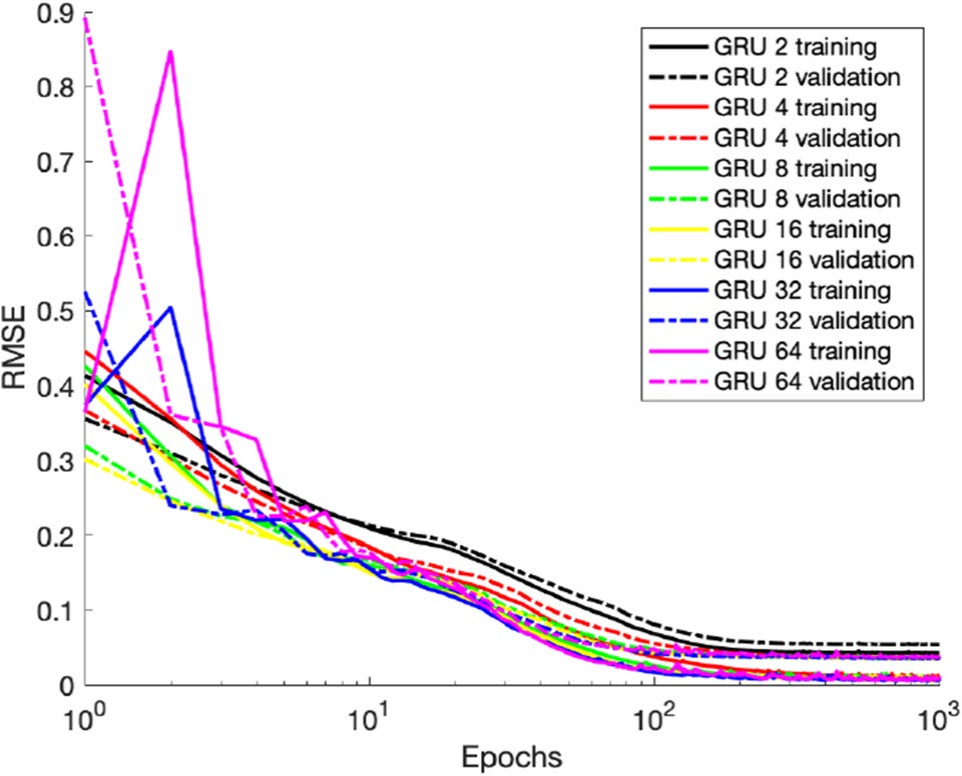
Average training and validation RMSE of ten runs, for 6 different hidden sizes of the GRU-based recurrent layer, for the iteration with the smallest validation loss.

**Figure 8:**
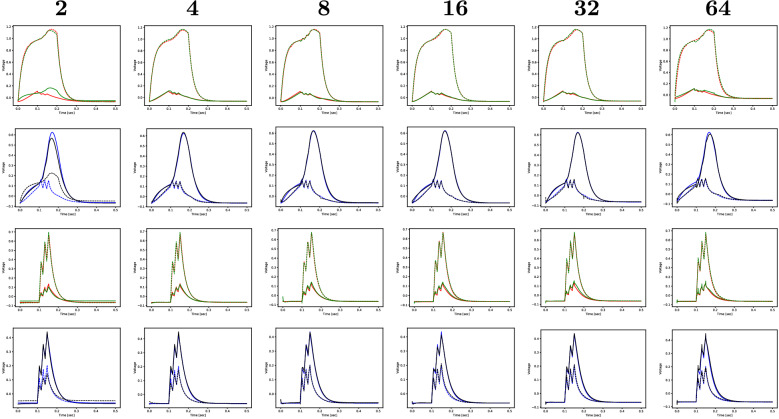
Experiment 2: realistic (red and blue) and predicted (green and black) sequences for DB1 and LUAL (1st and 3rd rows) and PVR and VB1 (2nd and 4th rows) for two sequences of the test set (one selected simulation out of ten).

We test both the LSTM and GRU units using the dataset with the coarser time step of (0.5 ms). Since the LSTM does not produce noticeable improvement over the GRU with a similar number of parameters, we only report here the results obtained with the GRU for six sizes of the recurrent layer: $2$, $4$, $8$, $16$, $32$, $64$ (Table 2). Figure 7 illustrates the evolution of the training and validation losses during the learning process, where one can see that for a size as small as 8 the model reaches a low and stable loss. In fact, from Fig. 8 and Table 2 we can state that a GRU with a size of 4 hidden units is optimal to reproduce the outputs with low RMSE.

**Figure 9:**
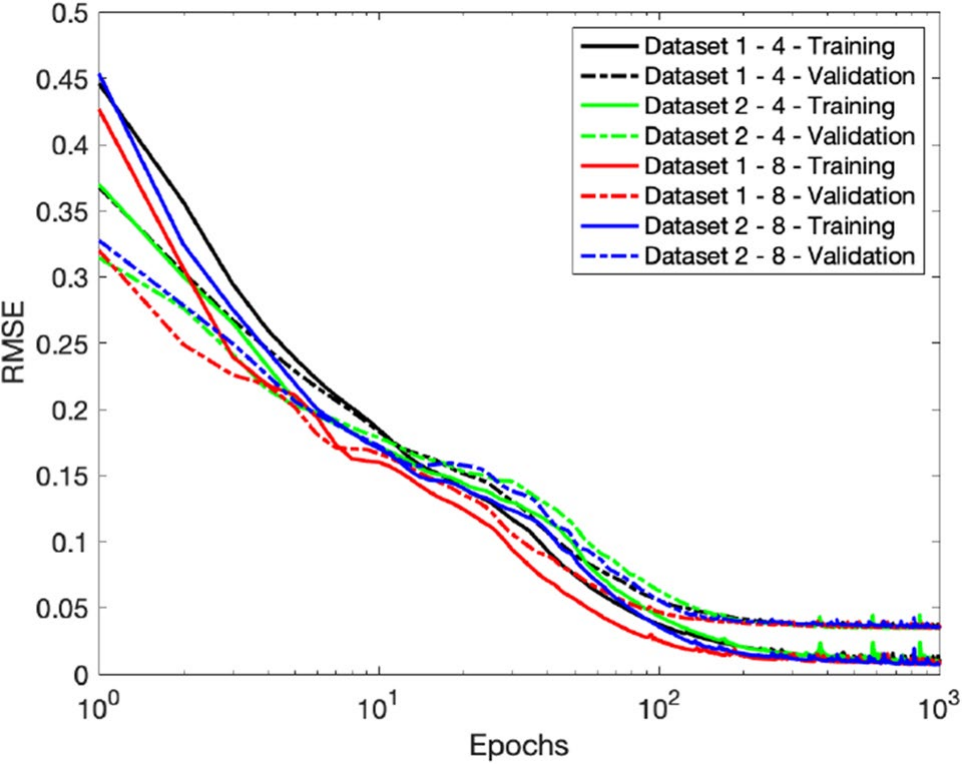
Average training and validation RMSE of ten runs, for the two datasets with a GRU-based recurrent layer with two different hidden sizes.

### Experiment 3: GRU (long sequences)

We now explore the models’ behaviour on data sampled with different time steps as in the same simulation interval this leads to sequences of different lengths. From a methodology standpoint this is important since even though time-wise the dynamics do not change, the temporal dependencies that the model has to learn are farther back in the sequence, which increases the difficulty of the learning process. We run the model for two datasets, one with the coarser (0.5 ms) time step and one with a finer (0.1 ms) one. The experiment is done only for the GRU, with $4$ and $8$ units.

**Table 3:** The average RMSE of ten simulations obtained with the GRU model, for the two datasets and two sizes of the recurrent layer (4 and 8 hidden units).

	Dataset 1–4	Dataset 2–4	Dataset 1–8	Dataset 2–8
Training	1.05e−02	1.08e−02	7.79e−03	8.17e−03
Validation	3.48e−02	3.45e−02	3.47e−02	3.54e−02
Test	1.17e−02	1.21e−02	1.00e−02	1.04e−02

**Figure 10:**
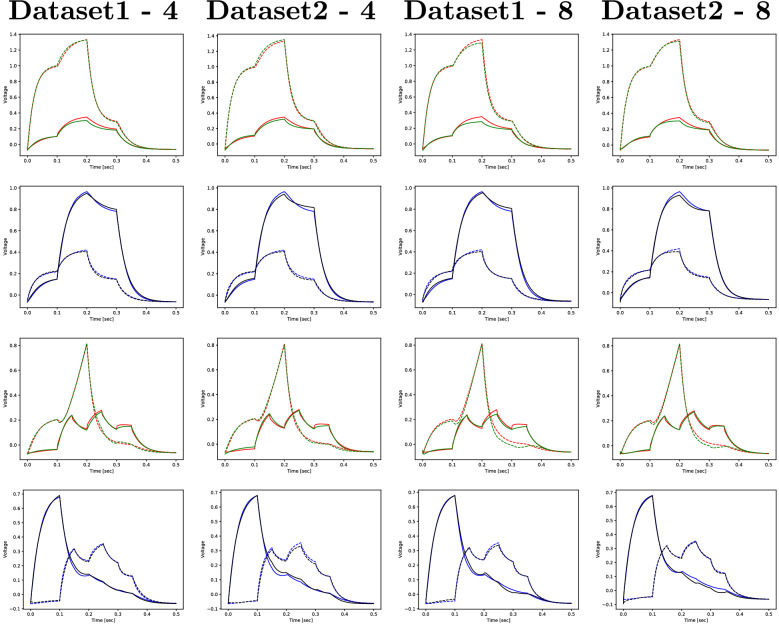
Experiment 3: realistic (red and blue) and predicted (green and black) sequences for DB1 and LUAL (1st and 3rd rows) and PVR and VB1 (2nd and 4th rows) for two sequences of the test set (one selected simulation out of ten).

Even though the model takes more time to converge for the finer time step, it ends up stabilizing with a loss of the same order in both cases and the model fits the test data well, as shown in Fig. 9 and Table 3. The plots in Fig. 10 further strengthen this idea.

### Experiment 4: LSTM & GRU (input importance)

Throughout this work we guaranteed the models are tested against unseen input signals, by deliberately placing in the test set data with inputs with unique and varied shapes. We now want to investigate the relative importance of certain inputs with respect to the outputs. Hence, in this experiment we train the models on the same simulation datasets as before, except we discard the data for two input neurons during the learning process, and see how the resulting models predict the outputs. We do this in two separate settings. First we train the models with recurrent layers of 8, 16 and 32, without the input information for the AVB neurons (No-AVB case). Next we repeat the process without the PLM data (No-PLM case).

**Figure 11:**
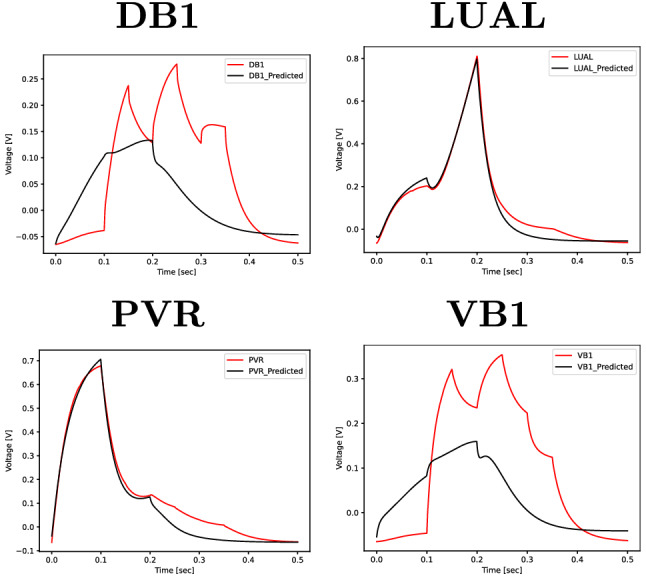
Experiment 4: realistic (red) and predicted (black) sequences for GRU-8 trained without input information of the AVB neurons for one sequence of the test set (one selected simulation out of ten).

The models perform similarly from an accuracy perspective and the size does not influence the overall RMSE. We notice that in each case, the models are able to accurately replicate the voltage of two output neurons but do a poor job at predicting the other two outputs. In the No-AVB case, the two neurons well reproduced are LUAL and PVR, as shown in Fig. 11, whereas in the No-PLM case the opposite occurs as the outputs of DB1 and VB1 are accurately reproduced while the other two exhibit a large RMSE. Table 4 shows the average RMSE of the two settings. In both cases the error reflects the models’ inability to accurately predict one pair of neurons. The larger absolute RMSE in one of the settings is merely a result of the increased voltage magnitude of LUAL and PVR neurons compared to DB1 and VB1 as in both cases the prediction is inaccurate for those respective nodes.

This result indicates that the AVB neurons are important for accurate prediction of the behaviour of LUAL and PVR, while the PLM inputs have more influence on the DB1 and VB1 neurons. This result is interesting from an interpretability standpoint, as it shows that some inputs are more relevant than others for specific outputs and behavioural scenarios.

**Table 4:** The average RMSE of ten simulations obtained with the LSTM and GRU with different sizes, when trained without input information of the AVB or PLM neurons.

	LSTM-8	LSTM-16	LSTM-32	GRU-8	GRU-16	GRU-32
No-AVB	6.74e−02	6.79e−02	6.81e−02	6.83e−02	6.79e−02	6.57e−02
No-PLM	2.00e−01	2.03e−01	2.01e−01	1.96e−01	1.96e−01	1.97e−01

### Experiment 5: LSTM & GRU (scalability and generalizability)

**Figure 12:**
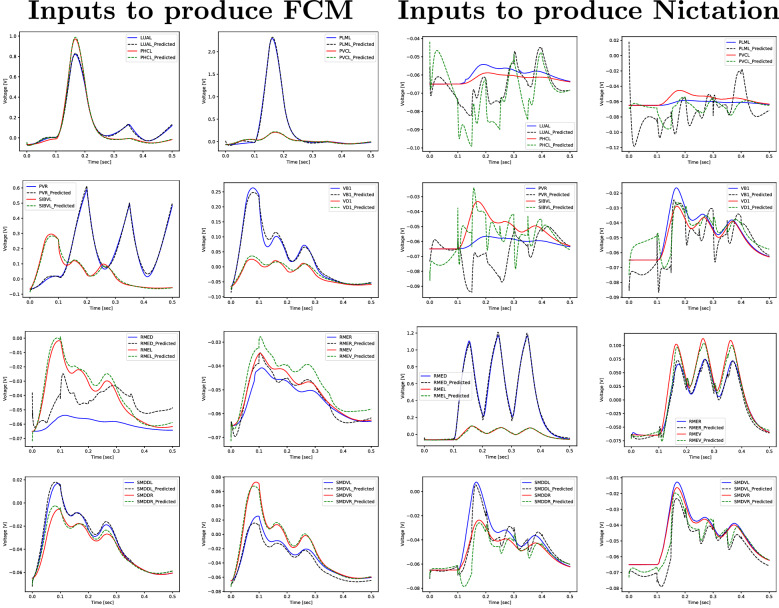
Experiment 5: realistic (red and blue) and predicted (green and black) sequences for various output neurons of the test set (one selected simulation out of ten) corresponding to FCM behaviour (first two rows) and Nictation behaviour (last two rows).

We create a dataset composed of data for two completely different behaviours, FCM and Nictation. For the FCM scenario the data is the same as described in “Data”, with the difference that we now record the responses of 16 output neurons instead of 4. In the Nictation case, we apply stimuli to 6 sensory neurons and we record 12 output neurons associated with neck and head muscles^11,13,46^. This new dataset therefore has a total of 80 examples, $4+6=10$ inputs and $16+12=28$ outputs. This experiment examines both the scalability potential of the resulting models, by significantly increasing the number of inputs and outputs, as well as their ability to predict more general data, coming from two different behaviours. LSTMs and GRUs with recurrent layer sizes of 8, 16 and 32 are trained on this data and the results are shown in Table 5. Both types of layers, and GRU in particular, are able to predict for a certain behaviour the output of the neurons of interest in that scenario, using a number of neurons for the recurrent layer inferior to the total of output neurons for which the voltage is predicted. This is apparent in Fig. 12, where we show various output sequences extracted from the test set for GRU with 16 hidden units. The model is indeed quite accurate overall in terms of RMSE, with some relative error showing for nodes where there is little to no activity, which is discarded since their response magnitude is within the absolute error metric used. However, this should not be an issue for most applications, as the important neurons for a given scenario are the ones with strong responses.

**Table 5:** The average RMSE of ten simulations for the dataset replicating the FCM and Nictation behaviours.

	LSTM-8	LSTM-16	LSTM-32	GRU-8	GRU-16	GRU-32
Avg RMSE	4.32e−02	3.96e−02	4.03e−02	3.74e−02	2.37e−02	2.63e−02

## Discussion

### Accuracy of the low-order model

Our analysis shows that a GRU with as low as 4 hidden units is consistently able to reproduce the outputs of the original model with a reasonably low RMSE and it adapts well to longer sequences, resulting from data sampled with both coarser and finer time steps. There is no noticeable difference in the errors between the two datasets in Experiment 3, the model consistently showing a RMSE below 1.3e-02 for the test set. What is interesting to note from Experiment 2 (see the RMSEs for Test in Table 2) is that increasing the number of hidden units to more than 16 worsens the predictions, so much that it is better to use a 4-units GRU than a 64-units GRU. As the RMSEs for the training sets are still decreasing for more units, this is probably due to overfitting of the data, a known problem in learning settings.

### Interpretability

We are interested in further understanding to what degree we are able to make some assumptions on the structure of the (reduced) model and extract a description, perhaps mathematical, for it. The fact that we are able to replace a complex biophysical model with a simpler recurrent neural network with few neurons also means the interpretability improves (helps to better understand the modelled neural circuits). Furthermore, in Experiment 4 we show that the models are able to accurately predict only certain outputs when deprived of certain inputs during training. This already suggests specific input-output dependencies, which a systematic study on input importance can further reveal and quantify. For a deeper analysis of what the RNNs learn, future work will focus on interpretability techniques, both model-agnostic and model-aware. Attention mechanisms and saliency maps can show the importance of inputs and features in the final prediction, which can further facilitate solving the inverse problem of extracting a small network from the RNN model for NEURON.

### Methodology

Generalizability indicates a (low-order) model’s ability to predict the original system response beyond the data used for modelling. From a machine learning perspective, this can be understood as prediction for a test set with input-output data unseen during training. While this is an expected merit of the neural networks in general and also demonstrated here, this is not the only implication of our modelling efforts. We are modelling a system having biophysical descriptions with well-established accuracy. However we are treating it as a black-box, using only peripheral input-output information from the initial high-fidelity simulations. Even though for many other more complex neural circuits the internal details are less known or completely unknown, this peripheral information can still potentially be obtained, hence the effectiveness of our models extends to these systems as well. This could especially be useful for specific cases (e.g. wet-labs extracting neural data from experiments on animals, or settings related to specific pathologies), where the system dynamics can be learned and predicted so that the number of real experiments further needed would be reduced.

## Conclusions

In this paper we create reduced order models for the *C. elegans* nervous system with three different recurrent neural networks architectures: simple RNNs, LSTMs and GRUs. The objective is to further generate a low-order description to replace the original, detailed model in the NEURON simulator. To achieve this goal we seek a model as simple as possible and therefore the ideal unit would appear to be the simple RNN. However, this unit does not perform sufficiently well compared to the other two architectures. The LSTM and GRU give comparable results in terms of overall fidelity, measured through RMSE, for different sizes of the recurrent layer. Due to its simplicity, GRU is preferable, and with a hidden size of 4 units, is able to reproduce with high fidelity, i.e. low RMSE, the original model’s responses to different types of stimuli. Furthermore, from a computational standpoint, explicitly inferring the response of the GRU model to such stimuli will vastly outperform the cost associated with simulating the high-fidelity model within NEURON, which has to solve the set of nonlinear equations implicit in the connectome network. Quantifying the potential advantage would require solving an inverse problem and performing an identical simulation of the extracted low-order model in NEURON, which is not the subject of this paper.

Further work will concentrate on improving the automation in choosing appropriate stimuli for the training, validation and test sets as well as optimal parameter selection. This will require a systematic analysis of compression possibilities of the learning-based models with error control. The novel concept of physics-informed machine learning will also help improving not only the predictions, but also the interpretability and generalizability of the neural nets. It implies adding structural or context information like physical constraints (domain, boundary conditions, initial conditions) to the training process. The resulting models will be more reliable, as they are guaranteed to satisfy physical laws, and they will potentially need less training data, making them even more suitable for experimental neuroscience, where there is a limited amount of labelled datasets available and in some cases it is not viable, for financial or ethical reasons, to procure additional data.

These results nonetheless show that it is feasible to develop recurrent neural network models able to infer input-output behaviours of realistic models of biological systems, enabling researchers to advance their understanding of these systems even in the absence of detailed level of connectivity.

## Data Availability

The datasets, models, the source code and the instructions to run them are available in the following GitHub repository: https://github.com/gmestre98/Celegans-ForwardCrawling-RNNs.
